# The Great Imitator: The Many Faces of Syphilitic Skin Presentations

**DOI:** 10.7759/cureus.59281

**Published:** 2024-04-29

**Authors:** Mohamed Bushry Basheer, Thajunnisha Mohamed Buhary, Dawn Friday

**Affiliations:** 1 Medicine, Cambridge University Hospitals National Health Service (NHS) Trust, Cambridge, GBR; 2 Genitourinary Medicine, London North West University Healthcare National Health Service (NHS) Trust, London, GBR

**Keywords:** men who have sex with men (msm), sexually transmitted infection (sti), maculo-papular rash, treponema pallidum, syphilis rash

## Abstract

We report the case of a 22-year-old heterosexual man presenting with a pruritic rash to the sexual health clinic. He was initially suspected of having a fungal rash by his general practitioner (GP) and treated with antifungals unsuccessfully. Subsequent testing revealed an active infection with *Treponema pallidum*. This was complicated by the concurrent fungal infection of the penile skin resulting in multiple lesions, requiring both antibiotic and antifungal treatment. With this case report, we hope to raise awareness amongst clinicians in non-traditional settings of the uncommon ways in which syphilis can present and to always consider it as a differential diagnosis, particularly in less likely populations.

## Introduction

Skin lesions in syphilitic infections are usually described as a maculopapular rash associated with a palmar-plantar involvement; they are usually not pruritic. However, less common patterns such as macular, pustular, and follicular (pruritic) rashes have also been described. In 2021, around 79% of syphilis cases were reported in those who are gay, bisexual and men who have sex with men (GBMSM) [1]. Diagnoses among the GBMSM community as well as heterosexual men and women have been increasing since 2010. In 2019, 21.5% of all syphilis diagnoses were reported in heterosexuals, with roughly equal distribution among heterosexual men and women [1].

Sexual histories may not be considered by general practitioners (GPs) for patients presenting with uncommon skin presentations of syphilis. This can result in misdiagnoses and unnecessary evolution of syphilitic infections. By presenting this case report, we hope to highlight the importance of widening the range of differential diagnoses through thorough history taking and raising awareness of uncommon presentations of serious pathology not commonly discovered in certain demographics.

## Case presentation

**Figure 1:**
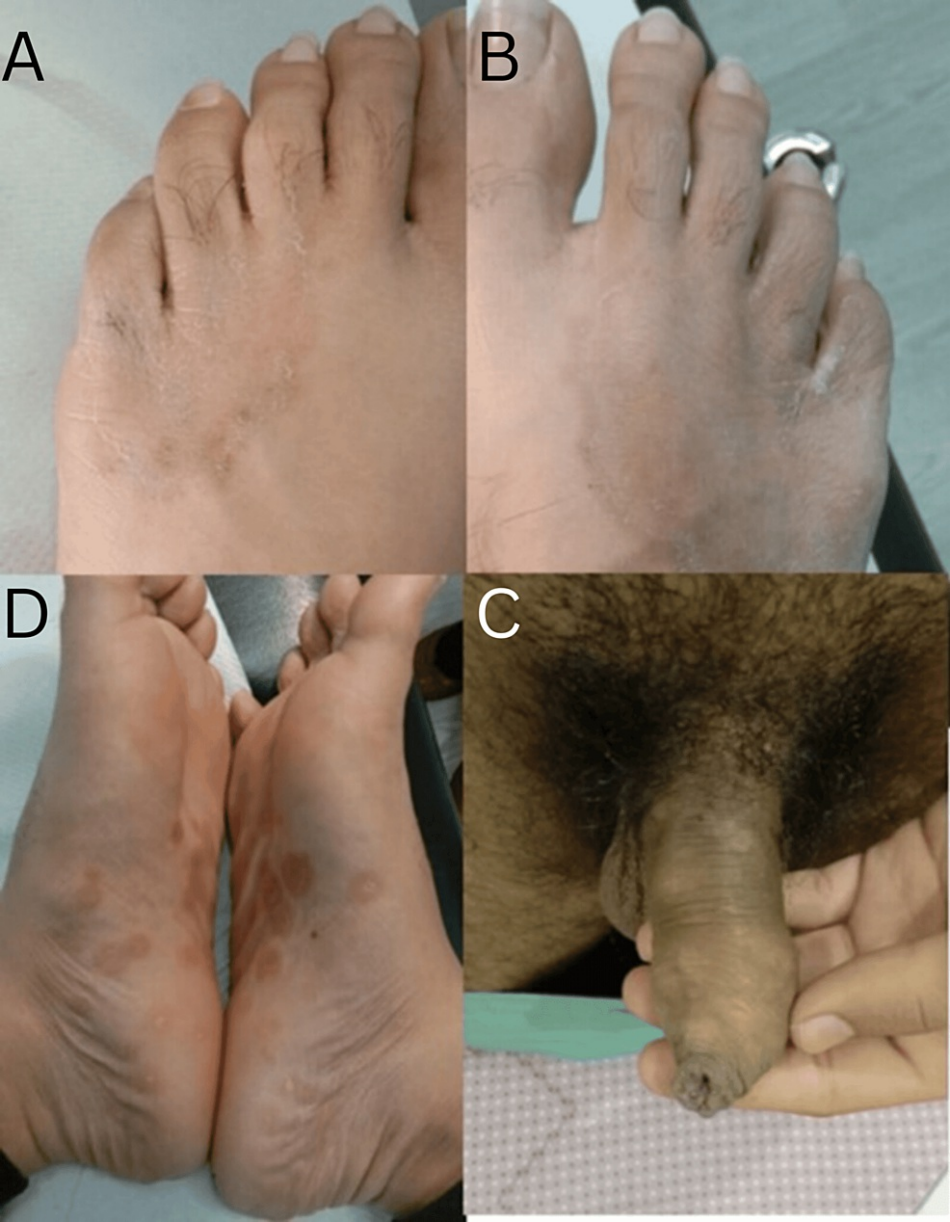
Images taken before treatment A: Papulosquamous rash in interdigital web spaces of the left foot; B: Spread of rash along the dorsum of the right foot; C: Rash consisting of well-defined scaly patches on the dorsum of the penile shaft; D: Image of both soles of feet showing well-defined, erythematous, scaly patches

A 22-year-old heterosexual man presented with a three-month history of pruritic, papulosquamous rash. It started in his feet in the interdigital web spaces (Figure 1A) and gradually spread to the dorsal and plantar surface (Figure 1D). After a few weeks, the rash appeared on the trunk and genital area. Both the papulosquamous and macular rash were pruritic. He had pharyngeal irritation at the beginning of the rash but no other constitutional symptoms. His GP suspected a fungal infection and therefore prescribed a local application of miconazole with 1% hydrocortisone cream, with no resolution of symptoms. His last sexual encounter was unprotected with a casual female partner four weeks prior to developing his symptoms. He also had two other sexual encounters, both females in the last three months. On examination, he had a faint, scattered, erythematous macular rash on his trunk, anteriorly and posteriorly. There were also well-defined scaly patches on the penile shaft (Figure 1C), scrotum, and both his soles (Figure 1D) and palms. A well-defined scaly rash was seen bilaterally on the lateral half of the dorsal surface of the foot (Figure 1B). The rashes on the plantar surface of the feet and the palmar surfaces were also mildly erythematous. 

Syphilis is becoming increasingly common in heterosexuals [2]. The rash on the dorsal surface of the right foot was not typical of secondary syphilis; nonetheless, we decided to test the patient for syphilis due to the palmar-plantar eruption pattern and the macular truncal rash prior to consideration of treatment. Serological testing showed the following: enzyme-linked immunoassay (ELISA) positive, *Treponema pallidum* particle agglutination assay (TPPA) of 1:1280, and rapid plasma reagin (RPR) of 1:64; thus confirming the diagnosis of syphilis. He was treated according to the British Association for Sexual Health and HIV (BASHH) [1] recommendations with one dose of benzathine penicillin 2.4 MU IM.

**Figure 2:**
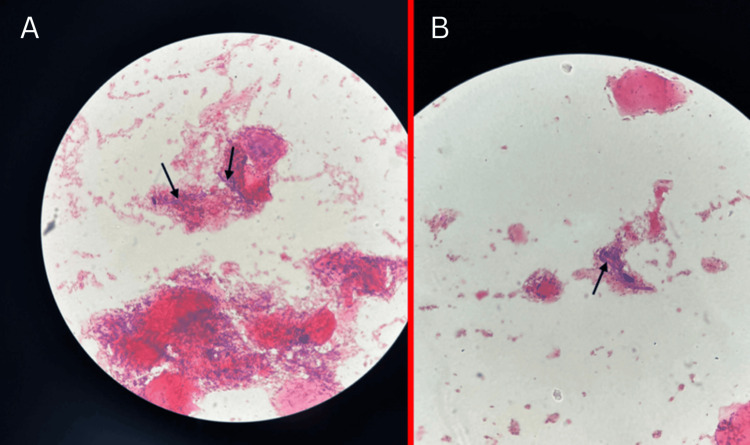
Gram stain microscopy Black arrows in panels A and B point to fungal filaments. The image shows Gram stain microscopy of skin scrapings taken from active margins of rashes on the dorsum of the patient's feet.

A review after two weeks showed the rash on the trunk, palms, and soles to have significantly improved, but not the rash on the scrotum and penile shaft. The rash on the dorsal surface of the feet was spreading and showed some active margins, suggestive of a fungal infection. Skin scrapings were taken from the active margins and the Gram stain showed fungal filaments (Figure 2). Therefore, he received itraconazole 200 mg twice daily for two weeks.

**Figure 3:**
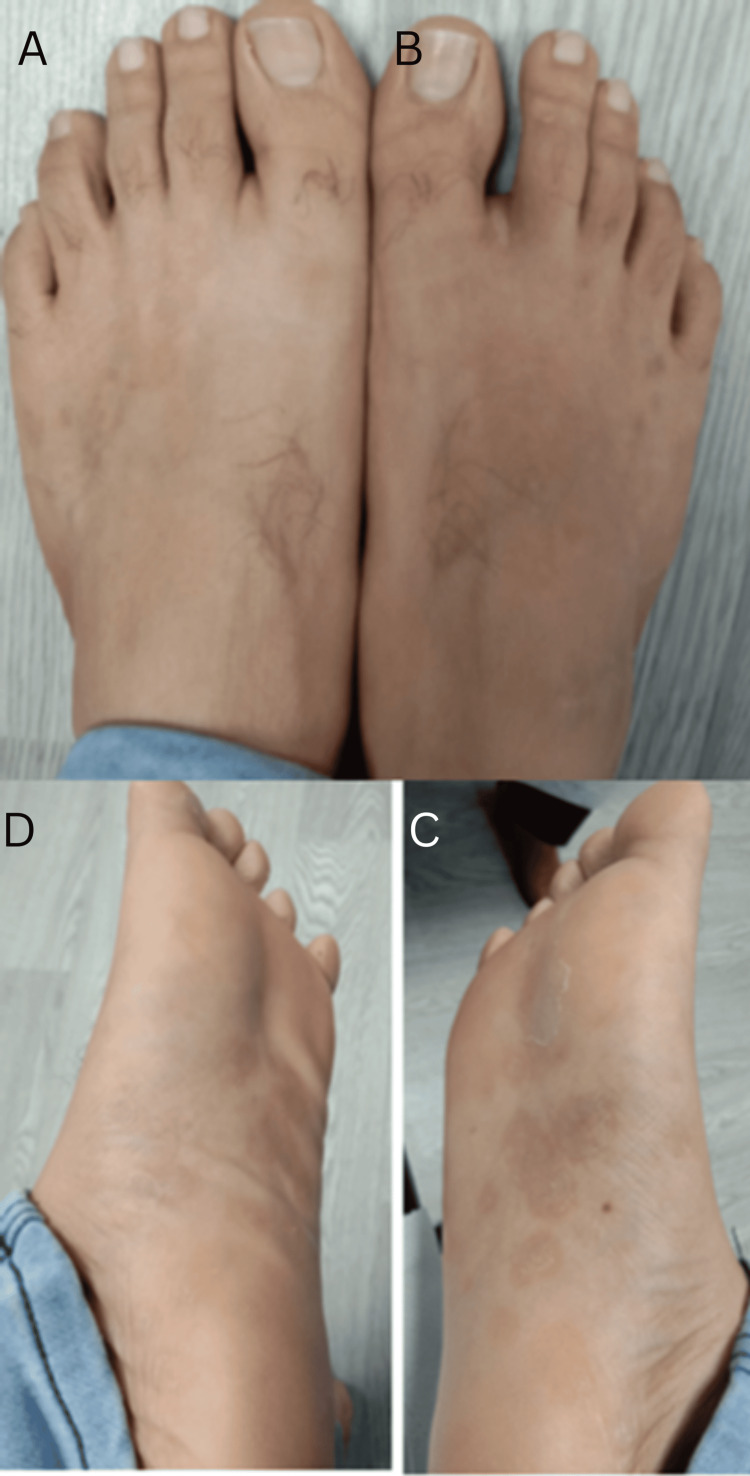
Images taken after treatment A marked improvement of the rash is seen in the dorsum of the left foot (A), dorsum of the right foot (B), plantar surface of the right foot (C), and plantar surface of the left foot (D).

The patient was followed up after two weeks and the rash on the feet (Figure 3) and genital skin showed marked improvement. His RPR dropped to 1:4 in the following six weeks indicating successful clearance of his infection. Other tests for sexually acquired infections, including HIV, were negative.

## Discussion

Syphilis is a chronic systemic infectious disease, and it has been known as ‘the great imitator’ due to its variable skin manifestation during the secondary stage. Secondary syphilis is the manifestation of disseminated *T. pallidum* infection. Constitutional symptoms can proceed and/or accompany dissemination, such as low-grade fever, malaise, sore throat, anorexia, patchy alopecia, headache, etc. If the headache is severe and persistent, it is most likely due to meningeal involvement. Around 75% of patients present with skin lesions that vary greatly and may resemble any generalized skin eruption (except vesicular or bullous); they can be macular/roseolar, papular, papulosquamous, or pustular [3]. Among these, the macular rash is the first to appear and the papular rash is the commonest and characteristic of secondary syphilis [3]. Clinicians should have a strong suspicion of secondary syphilis in patients presenting with widespread papulosquamous eruptions [4]. Papular eruption is characteristically and widely distributed over the trunk, arms, and legs. They are also found in palmar-plantar, facial, and genital patterns of distribution. It can give a sense of localized induration on palpation and usually is polymorphic. As a result of obliterative endarteritis and diminution of blood supply, scaling of the surface of the papules is common [5]. When the scaling lesions are predominant, the rash is described as ‘papulosquamous syphilide’. 

Papular lesions can become large and prominent, forming fleshy-looking masses called condylomata lata in intertriginous areas, and are the most infectious lesions of syphilis. The lesions can undergo central necrosis and present with a central core of necrotic tissue which may resemble a pustule. Occasional pustular lesions can be seen in papular or papulosquamous eruptions. Multiple pustular lesions are believed to occur in patients with malnutrition or certain comorbidities like uncontrolled diabetes.

A rare form of destructive syphilitic rash with deeply ulcerating lesions and toxemia which may be fatal if untreated is described in the literature as ‘malignant syphilis’ or ‘lues maligna’, with cases reported among patients infected with HIV [6-7]. There has been an increase in the use of pre-exposure prophylaxis (PrEP), a medication used to reduce HIV infection, among the GBMSM community and heterosexuals. This has resulted in a significant reduction in acquiring HIV infections. However, populations on PrEP have been associated with high rates of other sexually transmitted infections (STIs), in particular syphilis, and this is possibly due to high-risk sexual behaviors although the question of causation vs. correlation continues to be a topic of debate [8].

## Conclusions

Due to the increasing incidence of syphilis among heterosexuals, the diverse presentation of syphilis means it must be an important differential among sexually active individuals presenting in primary care and emergency settings. This is even more important given the increased use of PrEP among the GBMSM community and heterosexuals. Familiarity with syphilitic eruptions such as a palmar-plantar pattern and macular truncal rash will aid in considering this as a differential. Ensuring to note the patient's sexual history and checking whether the patient routinely tests for STIs in the context of rashes presenting in primary care will hopefully aid in earlier detection. We hope this case report will prompt readers to make such changes, if relevant, in their practice.
